# Vertebral Artery Hypoplasia in a Black Kenyan Population

**DOI:** 10.1155/2014/934510

**Published:** 2014-09-16

**Authors:** Julius Ogeng'o, Beda Olabu, Rankeet Sinkeet, Nafula M. Ogeng'o, Hemedi Elbusaid

**Affiliations:** Department of Human Anatomy, University of Nairobi, P.O. Box 30197, Nairobi 00100, Kenya

## Abstract

This study examined the characteristics of vertebral artery hypoplasia in 346 arteries of adult black Kenyans. The circumference was measured on haematoxylin/eosin stained microscopic sections of the distal one-third of the intracranial vertebral arteries using scion image analyser. Internal diameter was calculated in millimetre. Data were analysed using SPSS version 16.0. Vertebral artery hypoplasia (diameter < 2.0 mm) occurred in 100 (28.9%) arteries. Sixty of these (17.3%) were on the left and 40 (11.6%) on the right. Sixty (17.3%) were in females while 40 (11.6%) were in males. The side and gender differences were statistically significant at confidence interval of 95%. Frequency of vertebral artery hypoplasia was higher than in most other populations studied. The condition differs from that in other populations because it is more common on the left side and in females. We recommend ultrasound, angio-CT, or angio-MRI evaluation of vertebral arterial system before diagnostic or interventional procedures on posterior circulation.

## 1. Introduction

Vertebral artery hypoplasia (VAH) refers to those arteries with diameter of less than 2.0 mm [1–3]. This condition predisposes to posterior circulation stroke [4–7] and vertebral artery (VA) atherosclerosis [2, 8, 9] and can be confused with pathological occlusion from, say, atherosclerosis or dissection [10]. It is also associated deformities of other arterial components of posterior circulation including basilar and posterior communicating arteries [11, 12]. Characteristics of this condition are also important in selection and moulding of catheters during interventional neuroradiological procedures as well as mitigating complications of endovascular treatment and prognostication of cerebrovascular disease [11].

These characteristics of VAH show ethnic variation [13, 14]. As intracranial cerebral atherosclerosis becomes more common in Sub-Saharan African countries [15], there is need for data on African populations to inform management of disorders in posterior circulation. There are, however, currently few data from black African populations. This study, therefore, investigated the pattern of vertebral artery hypoplasia in an adult black population.

## 2. Materials and Methods

Two hundred and seventeen formalin fixed brains from adult black Kenyans obtained during autopsy at the Department of Human Anatomy, University of Nairobi, Kenya, were available for the study. Ethical approval was granted by the Kenyatta National Hospital/University of Nairobi Ethics and Research Committee. Forty-four were excluded—30 for suspected cerebrovascular disease and 14 for damage to the arteries. The cases of stroke and other cardiovascular disease were excluded to minimize the potential confounding effect of pathological conditions such as, for example, atherosclerotic, arterial narrowing. One hundred and seventy-three brains (99 males; 74 females, age range 20–79 years) were examined. Only cases of noncardiovascular death, as determined at autopsy, were included. The main causes of death were trauma (60.1%), infections (21.4%), malignancy (13.3%), poisoning (3.5%), and drowning (1.7%). The age distribution of the cases is as shown in Table 1.

The cardiovascular risk factors revealed included alcohol (32.3%), diabetes mellitus (23.4%), cigarette smoking (20.8%), and obesity (14.5%). The brains were divided into those of males and females. Arachnoid mater was gently peeled from the brainstem to expose vertebral and basilar arteries. Two millimetre specimens taken from the distal one-third of the intracranial vertebral artery were fixed in 10% formalin and processed for paraffin embedding and sectioning. Ten five micron serial sections from each arterial site were stained with haematoxylin/eosin and examined with light microscope at magnification ×40. The images taken by photomicroscope were digitized and internal circumference of each of the 10 sections from each site of the artery determined using scion^©^ image analyser. Only complete sections were included. Diameter in millimetres was calculated from the formula *D* = *C*/*π* where *D* is the diameter, *C* is the circumference, and *π* = 3.14. The average diameter of the 10 sections was taken to be the diameter of that artery. Vertebral artery hypoplasia was defined as internal diameter equal to or less than 2 mm. Photographs of representative samples of asymmetry were taken using a high resolution digital camera. Data were analysed using SPSS version 16.0 for Windows. Gender differences were analysed using the Student's *t*-test at 95% confidence intervals where *P* value ≤ 0.05 was taken as significant. Results are presented in digital macrographs, tables, and bar charts.

## 3. Results

**Figure 1:**
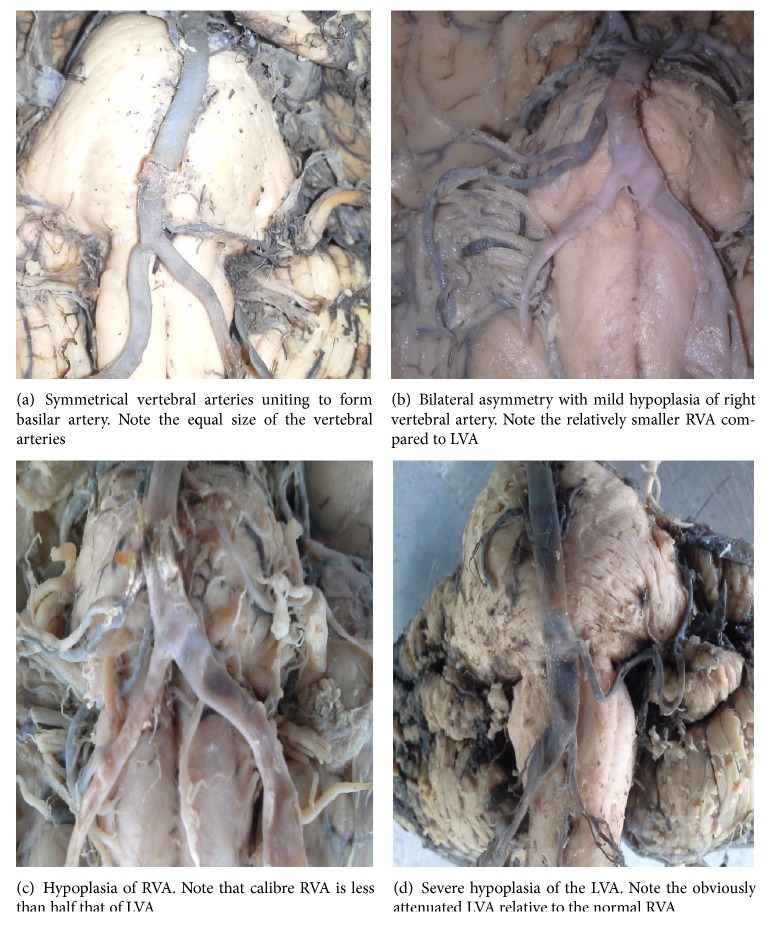
(a)–(d) Photomicrographs showing various grades of vertebral artery hypoplasia in an adult black Kenyan population. BA: basilar artery; VA: vertebral artery; LVA: left vertebral artery; RBA: right vertebral artery.

In all cases, two vertebral arteries joined to form the basilar artery. One hundred and two (29.5%) were symmetrical (Figure 1(a)), while 234 (70.5%) were asymmetrical, with right side dominance. The mean diameter for the right was 2.84 mm (±0.43 mm), while that for the left was 2.35 (±0.16 mm). This bilateral asymmetry was statistically significant (*P* = 0.047).

**Figure 2:**
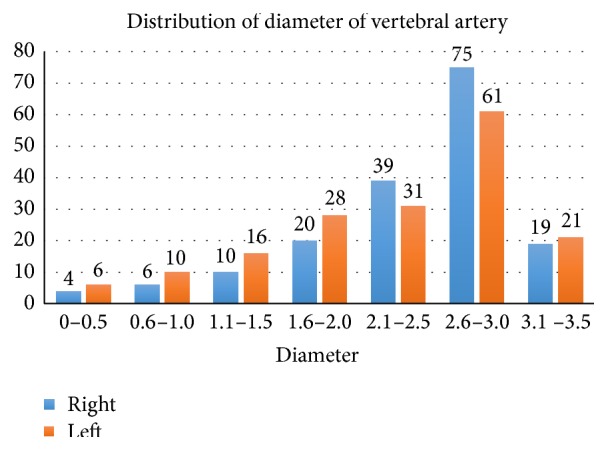
Distribution of diameter of vertebral artery in an adult black Kenyan population.

The overall diameter ranged from 0.4 mm to 3.4 mm with a mean of 2.65 (±0.16 mm). The mean diameter was higher in females (3.13 mm) than that in males (2.29 mm). The difference was statistically significant (*P* = 0.034). The peak diameter range was 2.6–3.0 mm, present in 136 (39.3%), followed by 2.1–2.5 mm (20.2%). Forty arteries (11.6%) measured >3 mm. Two hundred and forty-six (71.1%) were normal with diameter >2.0 mm. One hundred cases (28.9%) measured 2.0 mm and less; that is, they were hypoplastic (Figure 2). Of these hypoplastic ones 48 (13.9%) measured 1.6–2.0 mm; that is, they were mildly hypoplastic (Figures 1(b) and 1(c)). Twenty-six (7.5%) had a diameter of less than 1.0 mm and may be considered severely hypoplastic (Figure 1(d)). Twenty-eight of the hypoplastic arteries were left compared to 20 on the right. Sixty (17.3%) of the hypoplastic arteries were in males, while 40 (11.6%) were in females.

Among those with VAH, 60 were in males and 40 in females. The mean diameter for females was 3.14 mm, while that for males was 2.29 mm. The difference was statistically significant (*P* = 0.034) (Table 2).

## 4. Discussion

Observations of the current study that only 29.5% of vertebral arteries are symmetrical are consistent with the literature reports [16–18]. This asymmetry reflected in volume of blood flow has been related to origin of left subclavian artery directly from the aorta and angle of origin of vertebral artery [18]. Mean diameter of the vertebral artery of 2.65 mm is comparable to 2.6 mm reported for a study on an English population [19] but higher than 1.73 mm reported for a South African population [20]. It is lower than 3.25 for an Iranian population [18], 3.15 mm in Indian population [17], and 3.08 mm in Turkish [21] population.

These figures indicate wide variations in the diameter of vertebral arteries. Although such variations may be attributed to methodological factors, they probably reflect population differences [13].

The current study reveals a 28.9% prevalence of VAH, higher than 2.34–26.5% reported for most Caucasian [22–25] and Indo-Asian [2, 26–28] populations. Individuals with VAH have a high probability of posterior circulation stroke, with atherosclerotic susceptibility, ipsilateral lesions in the vertebral artery territory [2], and migraine with aura [29]. Indeed, even asymptomatic subjects with VAH have a significantly lower net VA flow volume and higher frequency of VA flow insufficiency [30]. Further, VAH serves as an independent factor of reduction of the posterior circulation blood flow velocity. It can also play a negative role in cases of occlusion of a major blood vessel since it limits potential compensatory blood circulation. In this way, it may lead to regional hypo-perfusion and complex neurovascular consequences which correspond to vestibular neuritis and migraine [3]. Accordingly, nearly 30% of the Kenyan population may be at risk of posterior circulation stroke and the other complications. This implies that in patients who present with vertebrobasilar insufficiency and aural migraine VAH should be considered.

The most remarkable observation of the present study is the left side predominance of VAH. This is at variance with most literature reports which show right side predominance [8, 9, 22, 28].

The present study reveals a male predominance, at variance with contemporary literature reports [5, 22, 31]. Although the studies may not be absolutely comparable because of differences in cutoff levels [3] and methodology, this diversity may reflect population variations probably based on epigenetic factors. Pertinent to this suggestions are assertions that the condition is hereditary [32, 33]. Accordingly, such population differences should be borne in mind during diagnostic and interventional neuroradiological and neurosurgical procedures on posterior circulation.

## 5. Conclusion

Vertebral artery hypoplasia occurs in about 30% of black Kenyans studied, higher than in most other populations studied. It also differs from other populations in that it is more common on the left side and in females. We recommend ultrasound, CT-angio or angio-MRI evaluation of vertebral arterial system before diagnostic or interventional neurosurgical and neuroradiological procedures on posterior circulation.

## Figures and Tables

**Table 1:** Age distribution of the population from which arteries were obtained.

Age range (years)	Frequency		Total [%]
	Male	Female	
21–30	15	11	26 [15]
31–40	19	15	34 [20]
41–50	26	19	45 [26]
51–60	25	18	43 [25]
61–70	10	9	19 [11]
71–80	4	2	6 [3]

Total	99	74	173 [100]

**Table 2:** Gender distribution of VAH among adult black Kenyans.

Range	Distribution	
	Male
0–0.5	6	4
0.6–1.0	10	6
1.1–1.5	16	8
1.6–2.0	28	22

Total	60	40
